# Searching for non-English literature may be unnecessary for German HTA Reports

**DOI:** 10.12688/f1000research.151365.2

**Published:** 2025-04-03

**Authors:** Elke Hausner, Sibylle Sturtz, Sandra Molnar, Lisa Schell, Wiebke Sieben, Stefan Sauerland

**Affiliations:** 1 Information Management Department, Institute for Quality and Efficiency in Health Care, Cologne, Siegburger Str. 237, Germany; 2 Department of Medical Biometry, Institute for Quality and Efficiency in Health Care, Cologne, Siegburger Str. 237, Germany; 3 Former employee: Department of Non-Drug Interventions, Institute for Quality and Efficiency in Health Care, Cologne, Germany; 4 Department of Non-Drug Interventions, Institute for Quality and Efficiency in Health Care, Cologne, Germany

**Keywords:** Language; Publication Bias; Publications; Retrospective Studies

## Abstract

**Background:**

Health technology assessment (HTA) reports are based on comprehensive information retrieval. Current standards discourage the use of search restrictions, such as publication date and language. Given limited resources, it was unclear whether the effort invested in screening and translating studies published in languages other than English provided relevant additional information compared with the inclusion of English-language publications alone. We therefore analysed the impact of non-English publications on the conclusions of HTA reports produced by the German HTA agency, the Institute for Quality and Efficiency in Health Care (IQWiG).

**Methods:**

We determined whether non-English publications were included in all German HTA reports on non-drug interventions (published by IQWiG between 06/2007 to 08/2018) and on selected drug interventions. If at least one non-English publication was included, we assessed for each endpoint whether or not the exclusion of non-English publications changed the conclusion. If a non-English publication did not contain information relevant to the HTA report, we classified the publication as “not relevant”.

**Results:**

Of 70 HTA reports, 38 (54%) included 126 non-English publications. In 4 reports (6%) with 50 endpoints investigated in 39 PICO questions, the exclusion of a total of 10 non-English publications led to a change in the conclusions for 13 endpoints (8 PICO questions). This was largely due to the fact that in many cases, non-English publications were the predominant or only literature available, resulting in a lack of analysable data after their exclusion.

**Conclusions:**

In general, studies only published in non-English languages have little influence on the conclusions of German HTA reports. For the vast majority of topics, a language restriction to English seems justified. Studies published in non-English languages may be useful in exceptional cases, for example when an intervention is only available in certain countries.

## Introduction

Health technology assessments (HTAs), which usually include systematic reviews, are based on comprehensive information retrieval requiring the use of multiple information sources. ^1^^,^^2^ Current standards discourage the use of search restrictions, such as publication date and language. ^1^^,^^3^ This is justified by the assumption that otherwise HTA conclusions could be biased, as studies with statistically significant results are more likely to be published in English-language journals, whereas non-significant results are more likely to be published in journals in languages other than English. ^4^ However, there is now evidence that significant results are increasingly being published in non-English journals. ^5^

In practice, many systematic reviewers restrict their searches to English-language articles. ^6^^,^^7^ For a long time, the reasons for this were of pragmatic nature: non-English literature is often more difficult to obtain and translation costs are high. ^8^ However, a recent systematic review by Dobrescu et al. ^9^ found out, that restricting evidence syntheses of interventions to English-language publications is a feasible methodological shortcut for most medical topics. The aim of our analysis was to examine whether the results of the systematic review are transferable to the context of HTA reports.

The Institute for Quality and Efficiency in Health Care (IQWiG ^10^) is a German HTA agency. In addition to more than 100 HTAs based on dossiers submitted by drug companies, IQWiG conducts around 10-15 other HTAs per year, mostly on non-drug interventions. The HTA reports investigate patient-relevant endpoints or validated surrogate endpoints and may comprise a number of different PICO questions on population, intervention, comparator, and outcomes. If possible, results of single studies are pooled in meta-analyses. The overall certainty of conclusions is graded into 3 levels of increasing certainty (hint, indication or proof of harm or benefit) according to the amount and quality of the available evidence. A detailed overview of IQWiG’s methods is provided in its methods paper. ^11^

IQWiG’s reports are generally based on comprehensive information retrieval. However, it was previously unclear whether it was worthwhile searching for, screening, and translating non-English publications on primary studies, i.e. whether their inclusion influenced the conclusions of the reports.

### Aim

The aim of this analysis was to assess the impact of non-English publications on the conclusions of German HTA reports.

## Methods

Following an internal project outline, we screened HTA reports (all reports on non-drug interventions published by IQWiG between 06/2007 and 08/2018 and randomly selected reports on drugs published between 09/2011 and 11/2016) for the inclusion of non-English publications. This procedure was not updated to include more recent HTA reports in the present article, as from September 2018 onwards, IQWiG restricted its searches to publications with English or German full texts. We focused on non-drug interventions, as we expected a higher number of non-English publications in this area.

In order to identify non-English publications, we screened all publications listed in the study pools of the HTA reports. This step was carried out via the EndNote databases of the underlying projects or, for older projects, via the reference lists in the HTA reports. In the next step, the language of the publications was identified using the entries in the bibliographic databases (MEDLINE, Embase). In a few cases this was not possible, so the language was checked using the full text or journal description. Data were extracted by one person and checked by another.

Only journal publications were included in the analysis, i.e., unpublished reports, conferences abstracts, evidence syntheses, clinical study reports, or registry entries were not considered. For updates of HTA reports, only the newly identified references were taken into account. For all HTA reports that included at least one non-English publication, we analysed whether the exclusion of such publications led to a change in conclusions; this was done separately for each PICO question and, if applicable, for each endpoint using specific categories ( Table 1).

**Table 1.** Classification of non-English publication.

Category number	Classification of non-English publication
A	Change in the conclusions of the HTA report
B	Conclusion does not change • Non-English study with low weight for PICO • All studies including non-English publication point into the same direction • Higher level of evidence compared to non-English publication available • Amount and quality of the evidence of all studies included in the HTA report was too low for reliable conclusions, regardless of inclusion of non-English publication • All studies included have unclear clinical relevance • Diagnostic study without direct relevance for the HTA report
C	Publication without relevance for the HTA report • Quality of study too low to change conclusion • A secondary publication in English was included • No data from non-English publication was used in the HTA report • Non-English publication was not translated

### Analysis

Using standardized methods (IQWiG’s methods paper ^11^ and internal guidance), we re-assessed the conclusions for the affected endpoints and PICO questions in the HTA reports. We did not re-analyse HTA reports on diagnostic test accuracy (DTA), as no conclusion on test accuracy endpoints is drawn in such reports.

For this purpose, the project manager reviewed all reports and assessed the impact of the exclusion of the non-English publication(s). This included the examination of individual studies on a specific endpoint or PICO question, qualitative re-assessments, and, in 3 cases, meta-analyses that had to be recalculated. When in doubt, the project manager consulted with another project manager or statistician.

For each endpoint, we defined a change in a conclusion due to the exclusion of non-English publications as either an upgrading or downgrading of the certainty of the conclusion or a complete loss of data (no conclusion possible). The effect of the exclusion of non-English publications on conclusions was categorized for each HTA report as follows (see Table 1): Category A: change in the conclusion for a particular endpoint (and therefore of the HTA report); Category B: no change in the conclusion for a particular endpoint (and therefore of the HTA report); Category C: the non-English publications do not contain relevant information for the HTA report (e.g. because the quality of the study was too low to change the conclusion) and are therefore classified as “not relevant”. The categorization was carried out independently by the project manager and a statistician. Any discrepancies were resolved by consensus between the two.

## Results

**Figure 1.**
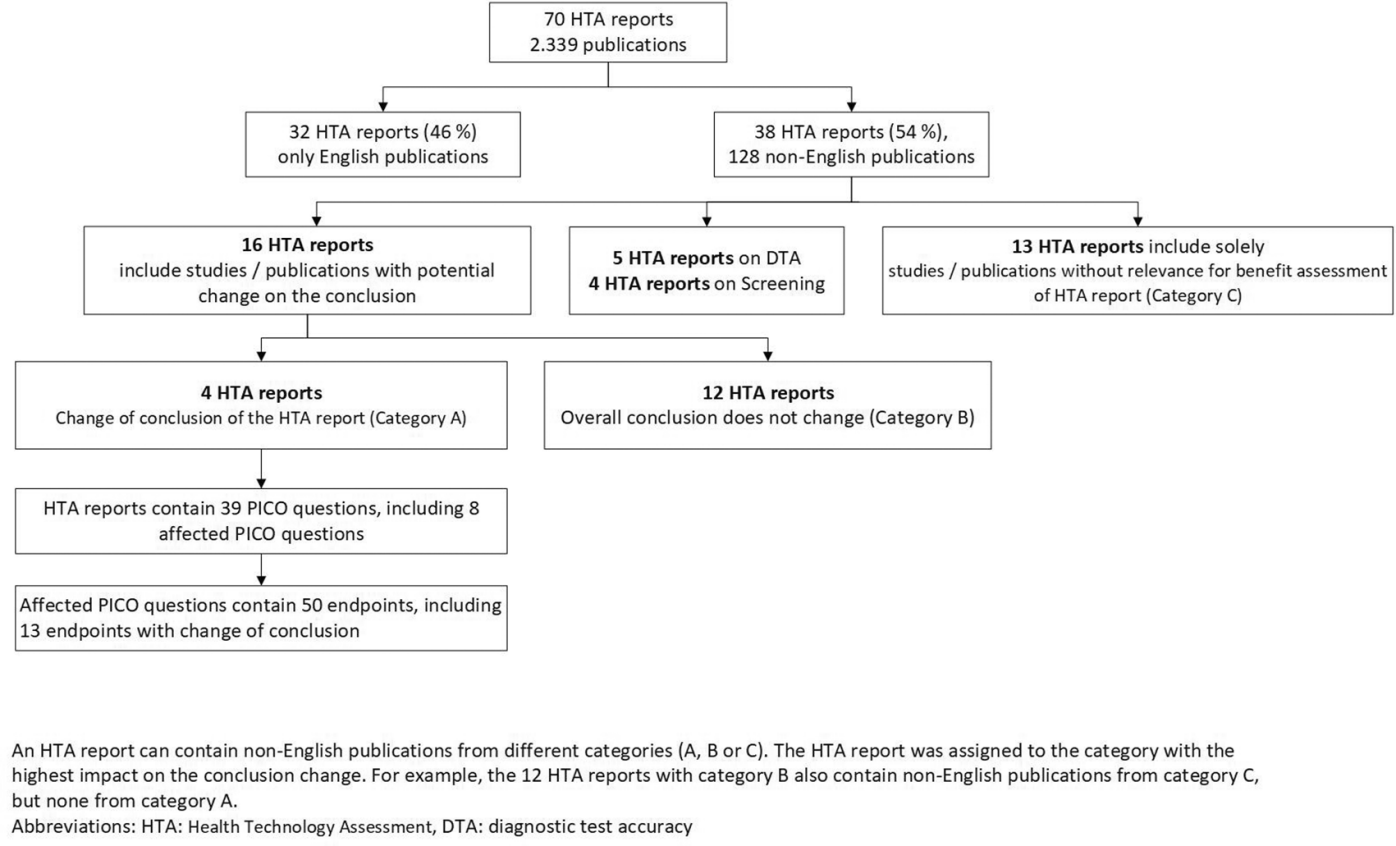
Flowchart on the presence and impact of non-English publications.

70 eligible HTA reports including 2328 publications were identified ^30^ ( Figure 1). Due to the inclusion of only a small number of selected drug reports, the vast majority of the HTA reports (96%) were on non-drug interventions ( Table 2).

**Table 2.** Characteristics of the HTA reports.

Characteristics of the HTA reports	Number (percentage) or median [minimum; maximum]
**Type of intervention** • Drug • Non-drug ○ Diagnostic ○ Screening ○ Treatment	3 (4) 67 (96) 16 (23) 16 (23) 35 (50)
**Number of publications included** • Number per report	2328 22.5 [1; 156]
**Design of studies included (defined a priori)** • RCTs only • RCTs + other study designs	33 (47) 37 (53)
**Number of bibliographic databases** **^a^**	3 [3; 23]

^a^ In addition, information retrieval for the HTA reports usually included a search of study registries and a check of reference lists of systematic reviews. Furthermore, requests to authors were made, if necessary, and public hearings took place (except for rapid reports).

Of the 70 HTA reports, 32 (46%) included English-language publications only ( Table 3). The remaining 38 reports (54%) included 126 non-English publications. These were most commonly published in German, Chinese, French and Spanish ( Table 4).

**Table 3.** Number of non-English publications included per HTA report.

Number of non-English publications included	Number of HTA reports
0	32
1	17
2	4
3-11	17

**Table 4.** Languages of non-English publications included in the HTA reports.

Language	Number (%) ^a^
German	41 (1.8%)
Chinese	30 (1.3%)
French	12 (0.5%)
Spanish	10 (0.4%)
other	33 (1.4%)

^a^ % of all included 2328 publications.

HTA reports with a change in the conclusions (Category A)

The 4 reports with a change in the conclusions were (short titles):

• A05-18: Tiotropium bromide for chronic obstructive pulmonary disease, ^12^^,^^13^
• N14-02: Systemic psychotherapy in adults ^14^^,^^15^
• N16-01: Active knee motion devices for anterior cruciate ligament ruptures ^16^^,^^17^
• N16-03: Continuous passive motion (CPM) devices after knee or shoulder surgery ^18^^,^^19^

For further details, see Table 5.

**Table 5.** HTA reports with endpoints that resulted in a change in the conclusions (Category A).

HTA reports	Title	Total number of PICO questions	Conclusion changed (PICO)	Studies published in non-English languages where exclusion from HTA report changed conclusions	Evaluated EP per affected PICO question	Conclusions changed (EP)
A05-18	Tiotropium bromide in the treatment of chronic obstructive pulmonary disease	12	2	Fang 2008 (Chi), Jia 2008 (Chi)	2	2
					9	1
N14-02	Systemic therapy in adults as a psychotherapeutic approach	18	4	Li 2010 (Chi), Yang 2005 (Chi), Wang 2011 (Chi), Wirsching 1989 (Ger), Cao 2007 (Chi), Zhang 2006 ^a^ (Chi)	2	2
					6	1
					17	1
					9	2
N16-01	Active knee motion devices in the treatment of anterior cruciate ligament ruptures	2	1	Von Lübken 2006 (Ger)	2	2
N16-03	Motor-driven continuous passive motion (CPM) devices after interventions on the knee and shoulder joint	7	1	Michael 2005 (Ger)	3	2

Abbreviations: Chi: Chinese; EP: endpoint; Ger: German.

^a^ 2 publications are available for the Zhang 2006 study: Zhang 2006a and Zhang 2006b.

These 4 reports investigated 50 endpoints in 39 PICO questions. In 3 out of the 4 reports (A05-18, N14-02, N16-01), the only available publication on a particular endpoint was a non-English publication. The exclusion of the non-English publication led to a change in the conclusion for 13 endpoints in 8 PICO questions ( Table 5). For 5 of these endpoints, the conclusion changed from a non-significant effect or an inconclusive result to “no conclusion possible” (due to a lack of data), and for 7 endpoints with previously statistically significant results, no conclusions could be drawn due to lack of data. For 1 endpoint, the conclusion changed from an inconclusive result to a hint of a benefit of the test intervention due to an effect becoming statistically significant (see example below).

Of the 10 studies that influenced the conclusions, 7 were published in Chinese and 3 in German ( Table 4).

*Example from an HTA report*

**Figure 2.**
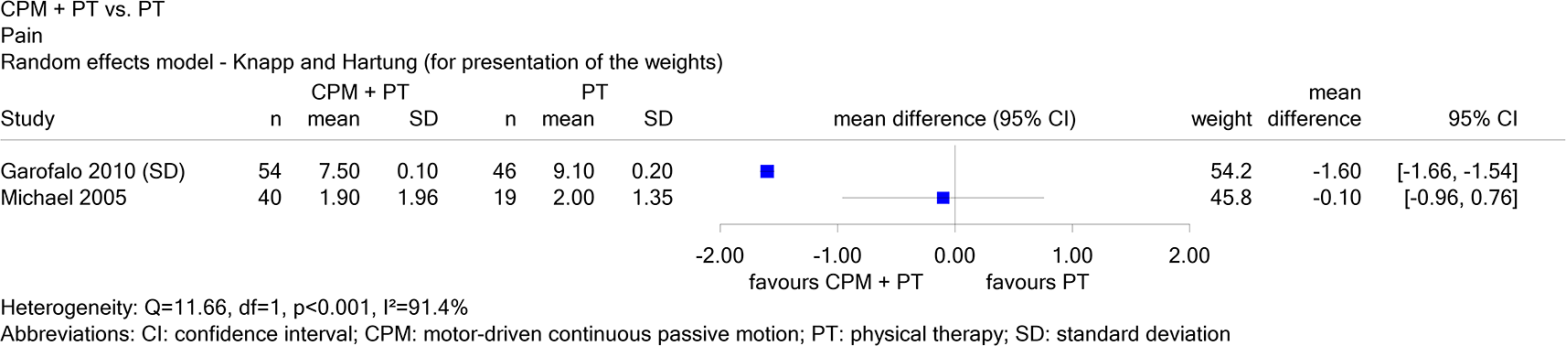
Report N16-03: Forest plot for the endpoint “pain”.

Report N16-03 investigated the use of continuous passive motion (CPM) devices after knee or shoulder surgery. For the comparison of shoulder devices in combination with physical therapy versus physical therapy alone in patients with rotator cuff rupture, there were 2 studies on the endpoint “pain”, one in English (Garofalo 2010) and one in German (Michael 2005). When both studies were considered, the results for this endpoint were inconsistent (see Figure 2), and therefore no conclusion could be drawn. When Michael 2005 was excluded, there was a hint of a benefit for combination therapy, i.e. the exclusion of non-English literature changed the conclusion.

HTA reports with no change in the conclusions (Category B)

If the exclusion of non-English publications did not change the conclusions of the HTA report, we identified the reason for this for each endpoint. In most cases, this was because there were several studies for each endpoint with results pointing in the same direction, meaning that the exclusion of a non-English publication had a negligible effect.

*Example from an HTA report*

**Figure 3.**
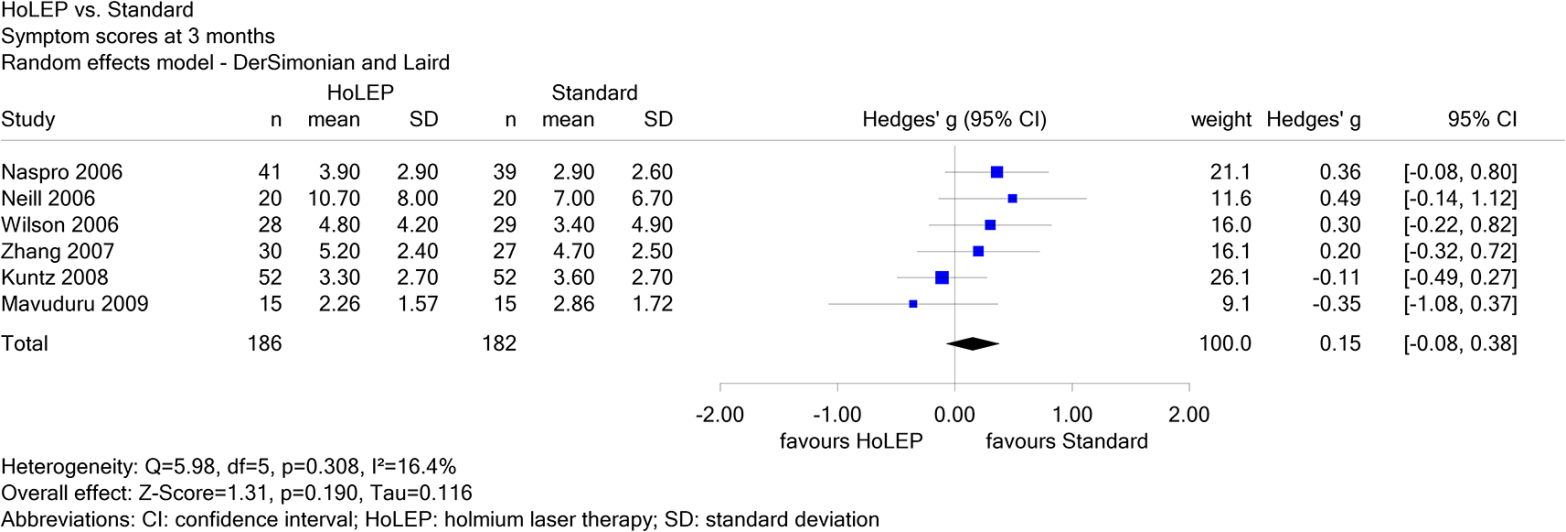
Report N09-01: Forest plot for the endpoint “symptom scores at 3 months”.

Report N09-01 investigated different non-drug local treatments in patients with benign prostatic syndrome. For the comparison of holmium laser therapy (HoLEP) versus standard treatment, 6 studies were included for the endpoint “symptom scores at 3 months”, of which one was in Chinese (Zhang 2007). Neither the result of a single study nor the pooled effect estimate showed a statistically significant effect. Zhang 2007 contributed a weight of 16.1% to the pooled effect estimate (see Figure 3); its exclusion did not change the conclusion.

Non-English publications classified as not relevant (Category C)

For the non-English publications assigned to Category C, the studies formally met the inclusion criteria of the HTA reports, and were therefore included in the study pool, but were not used in the actual assessment, mainly due to low quality of the study.

## Discussion

Our analysis shows that the exclusion of studies published in non-English languages has only a minor effect on the conclusions of HTA reports. This is consistent with the results of a recent systematic review by Dobrescu et al. on the restriction of systematic reviews of diagnostic or treatment interventions to English-language publications. They found that the inclusion of solely English-language publications led to a change in statistical significance in only 23 out of 259 meta-analyses (9%) and concluded that “the impact of restricting systematic reviews to English-language publications is negligible for most conventional medicine topics”. ^9^ Our analysis shows that the results can also be transferred to the context of HTA reports. In our analysis, most studies with an impact on the conclusions of the HTA reports were published in Chinese. This is notable, as the volume of Chinese-language literature is growing rapidly and there have been calls for the inclusion of such publications. ^20^^,^^21^ However, their quality has been questioned because of methodological flaws and overly positive results. ^5^

On the basis of our analysis, it seems reasonable to conduct additional searches for non-English literature if a particular intervention is exclusively or preferentially available in a specific region. For example, the “continuous active motion device” is a German product that is hardly marketed outside Germany. Only 2 studies could be identified for the HTA; both were published by German authors, one in English and one in German. This has less to do with a specific clinical question and more to do with market availability, which should of course be taken into account in preparation for the systematic search.

Language restrictions can save time. If an HTA report is being prepared under considerable time pressure, the exclusion of non-English literature should already take place at the level of the search strategy, resulting in a lower number of hits to be screened.

### Limitations

Methods for meta-analysis and for the assessment of study quality have changed over the past decades, which may influence the conclusions of HTA reports. We therefore tried to follow the original methods of the HTA reports included.

Only a small proportion of HTA reports required a recalculation of results.

At IQWiG, the analysis presented here led to the restriction of information retrieval in HTA reports to German- and English-language literature from September 2018 onwards (see the Methods section for details). This change in approach is also described in IQWiG’s updated methods paper. ^11^ For the present article, it was therefore not meaningful to update the pool of HTA reports by including HTA reports published by IQWiG after October 2019. An evaluation of current HTA reports from other large HTA agencies shows that they generally use a language restriction in their reports. ^22^^–^^29^

We did not analyse whether the non-English study publications were of lower quality than the English-language ones. However, all 10 non-English studies with change in the conclusions of the HTA report (see Table 5) were classified as highly biased in the underlying HTA reports.

## Conclusions

In general, studies only published in non-English languages have little influence on the conclusions of German HTA reports. For the vast majority of topics, a language restriction to English seems justified. Studies published in non-English languages may be useful in exceptional cases, for example when an intervention is only available in certain countries.

## Ethics and consent

Ethics and consent are not required.

## Data Availability

Zenodo: Searching for non-English literature may be unnecessary for HTA Reports - supplemental material (Version 3) [Data set]. https://doi.org/10.5281/zenodo.15025119. ^30^
▪ Supplementary-material_matrix_V3.xlsx (Data set of extracted references with details of the publication language) Data are available under the terms of the Creative Commons Attribution 4.0 International License (CC-BY 4.0). Zenodo: Searching for non-English literature may be unnecessary for HTA Reports - supplemental material [Data set]. https://doi.org/10.5281/zenodo.12642960. ^31^
▪ Supplementary-material_table.docx (HTA reports with endpoints that resulted in a change in the conclusion (Category A and B) Data are available under the terms of the Creative Commons Attribution 4.0 International License (CC-BY 4.0).
