# Supplementary material for: Searching for non-English literature may be unnecessary for German HTA Reports
Source: F1000Res. 2025 Jul 7;13:1134. Originally published 2024 Oct 4. [Version 3] doi: 10.12688/f1000research.151365.3 (PMC12012429; doi:10.12688/f1000research.151365.3)
Supplement: Peer review history

# Reviewer report: Reviewer response for version 3

**DOI:** 10.5256/f1000research.182757.r397138

**Published:** 2025-08-18

**Authors:** Tarquin Mittermayr

**Affiliations:** 1 Austrian Institute for Health Technology Assessments, Wien, Austria

Thank you for providing me with another opportunity to review the revised versions of this paper.

 I am happy with the way the authors have taken into consideration the reviewers' comments and the changes they have made accordingly. Furthermore, they have provided comprehensible arguments where they did not see a reason for changing the text.

 I agree with the convincing comment of another reviewer that this paper provides sound arguments for the readers to make their own assessment of the impact that limiting searches to contain only English-language publications may have.

Is the work clearly and accurately presented and does it cite the current literature?

Partly

If applicable, is the statistical analysis and its interpretation appropriate?

I cannot comment. A qualified statistician is required.

Are all the source data underlying the results available to ensure full reproducibility?

Yes

Is the study design appropriate and is the work technically sound?

Partly

Are the conclusions drawn adequately supported by the results?

Partly

Are sufficient details of methods and analysis provided to allow replication by others?

Partly

Reviewer Expertise:

Conducting systematic searches in the field of Evidence Based Medicine and Health Technology Assessment

I confirm that I have read this submission and believe that I have an appropriate level of expertise to confirm that it is of an acceptable scientific standard.

# Reviewer report: Reviewer response for version 2

**DOI:** 10.5256/f1000research.179748.r376060

**Published:** 2025-04-24

**Authors:** Liz Dennett

**Affiliations:** 1 University of Alberta, Edmonton, Alberta, Canada

This review reviewed 70 Health Technology Assessment  reports published at the Institute for Quality and Efficiency in Health Care in Germany between 2006 and 2018. They chose to mainly include (96%) non-drug HTA's because they felt those would be more likely to have non-english reports than the drug HTAs. Fifty four percent of these HTAs had english language studies included, but in only 4 HTA reports did the english language studies change results for any of the studies' endpoints.  In the 4 HTA reports, 10 studies changed the conclusions on 13 endpoints. The changes were not dramatic ones but in the case of 5 endpoints, no conclusion was possible without the non-english studies as they were the only studies containing information on that endpoint.  From these results the authors conclude: "In general, studies only published in non-English languages have little influence on the conclusions of German HTA reports. For the vast majority of topics, a language restriction to English seems justified". They continue to recommend searching for non-english studies in special cases (e.g. a device is only marketed in one country). 

 Overall, I believe this to be a well conducted study with transparent methods and it presents similar findings to a number of other published articles (all of the ones I am aware of are included in the Dobrescu, et al., 2021 (Ref 1) paper cited in this study. 

 I don't really have any comments that need to be addressed by the authors, but I can't help putting only a "Partly" for the question "Are the conclusions drawn adequately supported by the results? At the end of the day, you can't prove a negative, and it is impossible to definitively say, "It is completely fine to ignore non-english studies." This study does, however, add to the growing evidence that it is unlikely to matter that much. It is then up to individual teams if they are comfortable with the albeit small risk of losing important information for the feasibility trade off. When we train people to do evidence synthesis, we always remind students several times that the goal of every search is to be as comprehensive as possible. It is hard to go against that initial training, even when you come to appreciate that true comprehensiveness is rarely (if ever) possible.  I also think it is important to keep in mind the caveat (that these authors include) that China's growing influence in scientific discourse, which means that we can't consider this topic completely settled.

Is the work clearly and accurately presented and does it cite the current literature?

Yes

If applicable, is the statistical analysis and its interpretation appropriate?

Yes

Are all the source data underlying the results available to ensure full reproducibility?

Yes

Is the study design appropriate and is the work technically sound?

Yes

Are the conclusions drawn adequately supported by the results?

Partly

Are sufficient details of methods and analysis provided to allow replication by others?

Yes

Reviewer Expertise:

Expert searching

I confirm that I have read this submission and believe that I have an appropriate level of expertise to confirm that it is of an acceptable scientific standard.

## References

[1] : Restricting evidence syntheses of interventions to English-language publications is a viable methodological shortcut for most medical topics: a systematic review. *J Clin Epidemiol* .2021;137: 10.1016/j.jclinepi.2021.04.012 209-217 10.1016/j.jclinepi.2021.04.012 33933579

# Reviewer report: Reviewer response for version 2

**DOI:** 10.5256/f1000research.179748.r376055

**Published:** 2025-04-21

**Authors:** Steve McDonald

**Affiliations:** 1 School of Public Health and Preventive Medicine, Monash University, Clayton, Victoria, Australia

Thank you for the opportunity to review this revised version. This paper is a useful contribution to the evidence base of how language bias may affect the results of evidence synthesis. The authors provide a comprehensive case study of the impact of excluding non-English language publications on the conclusions of 70 HTA reports produced by IQWIG from 2007 to 2018.

 Previous peer reviewers raised several valid concerns and suggested amendments, notably that the conclusions of the study should be limited to the context of German HTA reports. I feel the authors have adequately addressed the concerns raised. The revised paper is well-written and clearly presented, with sufficient information provided to allow readers to make their own assessment of the value of restricting to the inclusion of English-language publications.

 The authors' conclusion that non-English publications "have little influence" or "minor effect" is supported by the data. Further, the authors provide a useful caveat to when searching for (and including) non-English publications should be considered.

 One brief comment: the analysis focuses on journal publications (which is reasonable). It is not clear if (for these 70 HTA reports) non-journal publications in languages other than English were included, and thus whether they had any impact on the the conclusions. I guess the question is to what extent the 'unnecessary' searching of non-English publications extends to non-journal article formats (conference abstracts, register entries).

 One minor comment: the flow diagram (second row) has 128 non-English publications, but in the Abstract and Results this is 126.

Is the work clearly and accurately presented and does it cite the current literature?

Yes

If applicable, is the statistical analysis and its interpretation appropriate?

Yes

Are all the source data underlying the results available to ensure full reproducibility?

Yes

Is the study design appropriate and is the work technically sound?

Yes

Are the conclusions drawn adequately supported by the results?

Yes

Are sufficient details of methods and analysis provided to allow replication by others?

Yes

Reviewer Expertise:

Information specialist with expertise in evidence synthesis.

I confirm that I have read this submission and believe that I have an appropriate level of expertise to confirm that it is of an acceptable scientific standard.

## References

[1] : Searching for non-English literature may be unnecessary for German HTA Reports. *F1000Research* .2025;13: 10.12688/f1000research.151365.2 10.12688/f1000research.151365.2 PMC1201242940265047

# Reviewer report: Reviewer response for version 1

**DOI:** 10.5256/f1000research.166008.r344992

**Published:** 2024-12-28

**Authors:** Tarquin Mittermayr

**Affiliations:** 1 Austrian Institute for Health Technology Assessments, Wien, Austria

I am happy to be invited to write a review on this relevant topic.

 Whilst the method for assessing the impact of excluding non-english literature on the conclusion of HTA reports is generally explained in a clear and comprehensible manner, Hausner et al fall short of explaining or referencing the internal protocol mentioned in the methods section.

 It would have also been helpful to either provide an explanation already in this section or refer to the appropriate page (e.g. “please find details on page 8”) in the limitations section as to why no publications after 2018 were considered for this assessment.

 Table 1 would benefit from clearer and more concise information:

 The same reasons for classifying low quality studies as category B in the classification table (“Amount and quality of the evidence…”) are listed in the explanation of the categories below the table under category C, thus giving room for ambiguous interpretations.

 Table 2 contains the total number of HTA reports included as well as the number per report. It is unclear if the number given for the latter (22.5) is an average, median or a percentage. In consideration of whether providing this number is meaningful, stating just the range of included publications per report might suffice and be more explanatory.

 The discussion section contains the confusing remark “We did not analyse whether the non-English study publications were of lower quality than the English language ones”. This appears to be contradictory to the explanations given for the classifications in Table 1. Should the contradictory nature of this statement be due to an imprecision in terminology (quality of evidence vs. quality of study publications), this study by Hausner et al would have benefited from performing a qualitative analyses of the publications themselves (in addition to analysing the containing evidence).

 In an article which argues that excluding non-English literature may have little impact on the overall conclusion of an HTA report, the example from Report N16-03 given in the Results section, where the exclusion of a non-English article led to a change in the conclusion, might sit uncomfortably with systematic evidence based medicine professionals. As graphical this example may be in showing the effect of this exclusion, it also appears to undermine the core argument of this article.

 A revision by the authors of this publication should focus on the coherence of their argumentation on the one hand, and on supporting their methods with more concise examples (e.g. Tables and Figures).

Is the work clearly and accurately presented and does it cite the current literature?

Partly

If applicable, is the statistical analysis and its interpretation appropriate?

I cannot comment. A qualified statistician is required.

Are all the source data underlying the results available to ensure full reproducibility?

Yes

Is the study design appropriate and is the work technically sound?

Partly

Are the conclusions drawn adequately supported by the results?

Partly

Are sufficient details of methods and analysis provided to allow replication by others?

Partly

Reviewer Expertise:

Conducting systematic searches in my position as an Information Specialist

I confirm that I have read this submission and believe that I have an appropriate level of expertise to confirm that it is of an acceptable scientific standard, however I have significant reservations, as outlined above.

# Response

**Published:** 2025-03-21

**Authors:** Elke Hausner

**Affiliations:** IQWiG, Germany

Thank you for your very helpful comments. We address all your points below and have revised the relevant sections in the manuscript.

 1. The reviewer indicates that it would be helpful to explain or reference the internal protocol mentioned in the methods section.

 Reply: We have exchanged the term “protocol” with “project outline”. This document is a German-language 1-page text that provides an overview of the project, not a complete step-by-step protocol. It was not published and we therefore cannot reference it.

**Change in the text: **We have amended the text

*“Following an internal project outline, we screened HTA reports (all reports on non-drug interventions published by IQWiG between 06/2007 and 08/2018 and randomly selected reports on drugs published between 09/2011 and 11/2016) for the inclusion of non-English publications.”*

 2. The reviewer states that it would have been helpful to either provide an explanation already in this section or refer to the appropriate page (e.g. “please find details on page 8”) in the limitations section as to why no publications after 2018 were considered for this assessment.

**Change in the text: **We have amended the text

*”For the present article, it was therefore not meaningful to update the pool of HTA reports by including HTA reports published by IQWiG after October 2019 (see the methods section for details).“*

 3. The reviewer states that table 1 would benefit from clearer and more concise information: The same reasons for classifying low quality studies as category B in the classification table (“Amount and quality of the evidence…”) are listed in the explanation of the categories below the table under category C, thus giving room for ambiguous interpretations.

 Reply: We disagree that there is ambiguity, because in category B the reason is “Amount and quality of the evidence of all studies”, whereas in category C the reason is “Quality of study too low to change conclusion”. The first reason refers to the evidence in its totality, the second reason addresses the quality of only the non-English study.

**No change to the text**

 4. The reviewer points out that in Table 2 it is unclear whether the number of included publications per report (22.5) is an average, median or percentage.

 Reply: Numbers and percentages are indicated by round brackets, while median and ranges are reported with square brackets. The heading indicates that 22.5 [1; 156] is the median, with the range given. We believe that it is reasonable to display median and range, rather than just reporting the range.

**No change to the text**

 5. The reviewer refers to the different information from Table 1 and the section ‘Limitations’ regarding the examination of study quality. In addition, the reviewer states that the study would have benefited from performing a qualitative analysis for publications.

 Reply: We are uncertain what type of “qualitative analysis” could be useful to be performed in addition to analysing the available evidence. As HTA work is evidence-based, the methods and the results of the primary studies are the sole data sources. Unfortunately, a qualitative analysis was not possible due to the limited time available. However, this would be very interesting, as there are already several publications [1-3] on this topic that question the quality of non-English studies.

**Change in the text**: We have added the assessment of the risk of bias for the non-English studies in the text.

*“However, all 10 non-English studies that changed the conclusions of an HTA report (see Table 5) were classified as highly biased in the underlying HTA reports.”*

 6. The reviewer points out that the example of N16-03 listed under ‘Results’ could lead to discomfort among experts in evidence-based medicine since it seems to undermine the core argument of this article.

 Reply: Admittedly, the recommendation not to search for non-English literature would have been easier if the decision to include or exclude such sources had not affected the conclusion of any HTA report. As 4 reports (6%) experienced a change in the conclusions, our conclusion is already somewhat toned down (“seems justified”). We agree with the reviewer that the decision is not straightforward. Some experts will continue to search non-English sources, because they consider the risk of overlooking a study more important than the resources required for searching for non-English publications. In our view, however, the main conclusion is correctly inferred from our results.

**No change to the text**

# Reviewer report: Reviewer response for version 1

**DOI:** 10.5256/f1000research.166008.r344994

**Published:** 2024-12-14

**Authors:** Sonia Garcia Gonzalez-Moral

**Affiliations:** 1 Newcastle University, Newcastle upon Tyne, England, UK

I welcome the opportunity to comment on this paper. With this study the authors have challenged the status quo of current systematic literature review guidelines such as those from Cochrane and health technology assessment methods recommendations for undertaking literature searches such as those recommended by EUneHTA. Such bold move feels justified if one understand the intensity of resource and capacity involved in HTA and so, while I support the rationale for this study and think that this is a good first attempt to breach this topic, in general I do not think that this study boasts the sufficient methodological rigor nor a big enough sample size to draw conclusions that could be generalized to HTA.

 To start with, the title of this manuscript *‘Searching for non-English literature may be unnecessary for HTA reports’* suggests the opposite of what the authors conclude ‘ *At IQWiG, the analysis presented here led to the restriction of information retrieval in HTA reports to German- and English-language literature from September 2018 onwards’.* I would therefore recommend that the title is revised, contextualizing this study to the needs and experience of the German HTA agency may increase the accuracy of their findings and conclusions.

 In the Introduction section the authors draw from one single study (Wu et al., 2009) to justify why non-English studies should no longer be searched for in HTA reports. Wu et al. analysed how randomised controlled trials were being reported in the Chinese literature written in Chinese. Their paper seemed to suggest that significant results were being published in languages other than English (in their case Chinese) and that RCTs were often poorly reported in Chinese. However, the authors of this manuscript later in the Discussion counterargue this point by remarking that ‘ *[…] as the volume of Chinese-language literature is growing rapidly and there have been calls for the inclusion of such publications. However, their quality has been questioned because of methodological flaws and overly positive results.*’ Deviating from the point of publication bias and focusing on the quality of the research as a reason for not searching non- English language publications in HTA. While the quality of research is extremely important, there are instruments that deal with that issue within the review process making ‘poor quality’ (according to Wu’s study) not *a priory* reason for not searching the entire scientific literature written on a particular language such as Chinese. In light of this contradiction the rationale for this study should be reformulated to accurately justify the reasons for not searching non-English papers and always placing these in the context of the German HTA agency. So far, the reasons presented here do not seem to warrant such claim.

 There are a number of omissions and inaccuracies through the methods section such as the search period in the abstract is not in line with the period reported in the Methods rendering this point unclear. Further, having read the full paper twice, I am still unsure of whether HTA reports of drugs were or not included in their assessment. I think the authors should provide a detailed list of the HTA reports (medicines and non-medicines) that were screened for inclusion in the methods section since these are not their results but the body of data they used for analysis. They should also justify why diagnostic HTA reports would have been included from the start if they were not to be re-analysed in the results. Furthermore, the authors seem to apply the inclusion and exclusion criteria unsystematically when they declare that they only considered journal articles cited in HTA reports but then they present as a limitation that ‘ *For 2 reports (D06-01B and D06-01C), we also included evidence syntheses, although this document type was to be excluded.’* But fail to provide an explanation as to why such allowance was made.

 In the results section two random examples are provided of HTA report, can the authors justify why these are explained in more detail and not the others? which criteria led the authors to provide these two examples? This feels selection bias and should be avoided.

 In the Discussion section authors seem to contradict themselves. They start by saying that *‘the exclusion of studies published in non-English languages has only a minor effect on the conclusions of HTA reports’* At this point it is important to note that the measure of such effect (whether is big or small) is not provided throughout the manuscript nor it is explained how this ‘change in effect’ was estimated, there are no statistical tests and no p values provided making is really difficult to study whether the change, although minor, might or might not have been significant for the results of the HTA report and their future recommendations. The section follows by saying that ‘ *On the basis of our analysis, it is not possible to identify topics where non-English publications might be relevant.’* This statement seems to contradict what has just been said. And finally, they acknowledge that actually *‘it seems reasonable to conduct additional searches for non-English literature if a particular intervention is exclusively or preferentially available in a specific region’.* The latter being a statement which defeats the point of the entire manuscript in its current rationale and supporting evidence.

 Please fix reference number 1 to reflect the title (Cochrane Handbook for Systematic Reviews of Interventions) of the handbook in with Chapter 4 is included. Table 1 would benefit from including definitions for each of the categories. Figure 1 please include the distribution of the 38 reports as they are allocated to different categories. Table 5. What does EP mean?

Is the work clearly and accurately presented and does it cite the current literature?

No

If applicable, is the statistical analysis and its interpretation appropriate?

Not applicable

Are all the source data underlying the results available to ensure full reproducibility?

Partly

Is the study design appropriate and is the work technically sound?

Partly

Are the conclusions drawn adequately supported by the results?

No

Are sufficient details of methods and analysis provided to allow replication by others?

No

Reviewer Expertise:

Information retrieval, systematic reviews, HTA, health research methods

I confirm that I have read this submission and believe that I have an appropriate level of expertise to confirm that it is of an acceptable scientific standard, however I have significant reservations, as outlined above.

# Response

**Published:** 2025-03-21

**Authors:** Elke Hausner

**Affiliations:** IQWiG, Germany

Thank you for your very helpful comments. We address all your points below and have revised the relevant sections in the manuscript.

 1. The first point raised is that the study is not suitable for making general statements about HTA reports.

 Reply: This point was also raised by Reviewer 1 (see above). We have taken your concerns and suggestions and adapted the text

**Change in the text: **instead of referring to HTA reports in general, the text now refers specifically to the German HTA reports

 2. It was also criticized that the authors draw from one single study (Wu et al., 2009) to justify why non-English studies should no longer be searched for in HTA reports.

 Reply: This is a misunderstanding, as Wu 2009 was cited primarily to show that the common assumption that foreign-language articles primarily produce negative/neutral results should be questioned. In order to strengthen the rationale for why we see our study as an important contribution, we have added a current systematic review in the background section, which we had originally only mentioned in the discussion.

**Change in the text:** In the background section, we have added the systematic review by Dobrescu et al. as a rationale for the present analysis. Please see changes in the text on page 4.

*“However, a recent systematic review by Dobrescu et al. [9] found, that restricting evidence syntheses of interventions to English-language publications is a feasible methodological shortcut for most medical topics. The aim of our analysis was to examine whether the results of the systematic review are transferable to the context of HTA reports.”*

 3. Another comment refers to different information on the search / publication period in the abstract and the methods section.

**Change to the text: **We have now presented the publication period in the abstract.

*“We determined whether non-English publications were included in all HTA reports on non-drug interventions (published by IQWiG between 06/2007 to 08/2018) and on selected drug interventions.”*

 4. It is also noted that it remains unclear whether HTA reports on drugs were included in the analysis.

 Reply: Table 2 displays the number of included drug / non-drug HTA reports (3 HTA reports on drugs, 67 on non-drugs).

**No change to the text**

 5. The reviewer recommends providing a detailed list of HTA reports (drug and non-drug) that were screened for inclusion in the methods section.

 Reply: As stated in the methods, we included all HTA reports on non-drug interventions (published between 06/2007 and 08/2018) and selected 3 reports on drugs. We have added a list of analysed HTA reports to the supplement.

**Change to the text: **We have added a list of included HTA reports https://doi.org/10.5281/zenodo.12642959

 6. The reviewer would like us to justify why diagnostic HTA reports were included from the beginning if they are not to be re-analysed in the results.

 Reply: HTA reports on diagnostic questions can contain both results on the benefit of the diagnostic measure and on the diagnostic accuracy. Publications in other languages than English only contained information on diagnostic accuracy. However, in no case was a conclusion derived from this, but only the diagnostic accuracy was reported. Our article focuses on the impact of non-English publications on the conclusions of HTA reports, therefore a comparison of conclusions based on different data sets is not possible.

**No change to the text**

 7. The reviewer is concerned that the inclusion and exclusion criteria were applied unsystematically and asks for an explanation as to why 2 HTA reports are cited under the limitations.

 Reply: Based on your concerns, we have revised the extraction of the included studies in the HTA reports and have deleted the entries that did not fulfil the inclusion criteria (n = 11).

**Change in the text:** The tables and figures in the text have been adjusted accordingly. The total number of references included is now 2328, the number of non-English publications 126. Thus, we have deleted the corresponding section in the limitations.

 8. In addition, the reviewer notes that there are two examples of HTA reports listed in the results section and asks for a more detailed explanation of why these were selected.

 Reply: We wanted to illustrate a little more clearly to the reader why a non-English language article was categorized as A or B. As these are only examples, we see no risk of bias in the selection. The full list of HTA reports with non-English articles in categories A and B can be found in the supplement (https://doi.org/10.5281/zenodo.12642959).

**No change to the text**

 9. The reviewer points out that the measure of effect is not explained and that no statistical tests and no p-values are given. In her view, this makes it difficult to analyse whether or not the change, even if small, could have been significant for the results of the HTA report and the future recommendations.

 Reply: The measure of effect was defined as “change in the conclusion of the HTA report” which is composed of the conclusions of the individual endpoints. An overall conclusion was not provided by the underlying HTA reports. For most HTA reports (94%) no changes in the endpoints were identified. All changes were assessed in a yes/no format. This effect measure was therefore qualitative, even it was partly based on statistical analyses and re-analyses. The reviewer rightly points out that even small changes in a meta-analysis may or may not have been significant for the results of the HTA report. For this very reason, our analyses were not based on any statistical comparison between primary analysis and re-analysis. Rather, we examined whether the re-analysis led to a change in the conclusion of the endpoint and thus the HTA report, as this determines reimbursement and availability of health care services.

**No change to the text**

 10. According to the reviewer, it is contradictory first to conclude that searching the non-English literature is generally unnecessary, but then to explain that such searches may be worthwhile for particular interventions that are available only a specific region.

 Reply: We agree that a single HTA report on a country-specific intervention (N16-01) is a weak basis for recommending additional searches of non-English sources under specific circumstances. However, our text only describes this option as “reasonable” – without recommending it for all clinical interventions without clear indications that this is useful in the respective case.

**Change in the text: **We amended the text in the discussion

*“This has less to do with a specific clinical question and more to do with market availability, which should of course be taken into account when preparing the systematic search.”*

 11. The reviewer asks to fix reference number 1 to reflect the title (Cochrane Handbook for Systematic Reviews of Interventions) of the handbook in which Chapter 4 is included.

**Change in the text:** We amended the citation

*1. Lefebvre C, Glanville J, Briscoe S et al. Chapter 4: Searching for and selecting studies [last updated September 2024]. In: Higgins JPT, Thomas J, Chandler J, Cumpston M, Li T, Page MJ, Welch VA (editors). Cochrane Handbook for Systematic Reviews of Interventions version 6.5 [online]. 2024 [Accessed: 20.01.2025]. URL: https://training.cochrane.org/handbook/current/chapter-04*.

 12. Table 1 would benefit from including definitions for each of the categories. You can find a description within the text (page 4): “Category A: change in the conclusion for a particular endpoint; Category B: no change in the conclusion for a particular endpoint; Category C: the non-English publications do not contain relevant information for the HTA report (e.g. because the amount or quality of the data is too low) and are therefore classified as “not relevant”.”

**Change in the text:** We have added the reference to Table 1 to the text

*“The effect of the exclusion of non-English publications on conclusions was categorized for each HTA report as follows (see Table 1)”*

 13. The reviewer asks to include in Figure 1 the distribution of the 38 reports as they are allocated to different categories.

 Reply: The requested information can be seen in Figure 1. As shown in the figure, 13 HTA reports contain only category C publications, 12 HTA reports with category B and 4 HTA reports to category A. The footnote also refers to the fact that an HTA report can contain non-English publications from different categories (A, B or C). The HTA report was assigned to the category that has the greatest impact on the change in conclusion. For example, the 12 HTA reports with category B also contain non-English publications from category C, but none from category A.

**No change to the text**

 14. The reviewer asks what the abbreviation EP in Table 5 means.

 Reply: This is the abbreviation for “endpoint“. An overview of the abbreviations used in the table is added below the table.

**No change to the text**

# Reviewer report: Reviewer response for version 1

**DOI:** 10.5256/f1000research.166008.r332664

**Published:** 2024-11-18

**Authors:** Knut Sundell

**Affiliations:** 1 Department of social work and criminology, University of Gävle, Gävle, Sweden

Different aspects of publication bias (positive outcome bias, time-lag bias, and place-of-publication bias) constitute serious threats to the validity of research synthesis. If research syntheses are based on a biased selection of the available research, there is an increased risk of producing misleading results. Awareness of these types of bias is important for reading systematic reviews and incorporating their findings into clinical practice and decision making.

 The authors have screened 70 HTA reports from the German HTA agency, the Institute for Quality and Efficiency in Health Care (IQWiG) searching for reports that include non-English articles. If at least one non-English publication was included, the authors assessed whether the exclusion of non-English publications changed the conclusion. Of 70 HTA reports, 38 (54%) included non-English publications. The exclusion of non-English studies has only a minor effect on the conclusions of HTA reports

 The authors conclude that, (1) *in general, studies published in non-English languages have little influence on the conclusions of HTA reports*, and (2) *For the vast majority of topics, a language restriction to English seems justified.*

 These conclusions seem premature because of possible selection bias. We don’t know if the non-English articles in these reports constitute all relevant articles or a skewed sample (e.g., because of positive outcome biased, ). To be able to conclude that non-English articles have little influence on the report conclusions, the articles need to represent all relevant articles (i.e., no publication bias) which will require an extensive search for grey literature, including non-peer reviewed articles, the types of articles that the authors have excluded (i.e., conferences abstracts, evidence syntheses, clinical study reports, or registry entries).

 The best we can say about the results is that non-English articles that are included in IQWiG’s reports may have had scant influence on the HTA-reports’ conclusions. This may be a thought-provoking result but hardly one to base policy on including or excluding non-English articles.

 Furthermore, I suggest that the authors discuss the types of bias and their relative importance for the design of the study. The exclusion of non-English publications might be motivated, but the results might still be biased because of positive outcome bias, time-lag bias, place-of-publication bias or other types of bias?

 There is no motivation for only including the German HTA organization. What are the pros and cons of this choice?

Is the work clearly and accurately presented and does it cite the current literature?

Yes

If applicable, is the statistical analysis and its interpretation appropriate?

I cannot comment. A qualified statistician is required.

Are all the source data underlying the results available to ensure full reproducibility?

Yes

Is the study design appropriate and is the work technically sound?

No

Are the conclusions drawn adequately supported by the results?

No

Are sufficient details of methods and analysis provided to allow replication by others?

Yes

Reviewer Expertise:

NA

I confirm that I have read this submission and believe that I have an appropriate level of expertise to state that I do not consider it to be of an acceptable scientific standard, for reasons outlined above.

# Response

**Published:** 2025-03-21

**Authors:** Elke Hausner

**Affiliations:** IQWiG, Germany

Thank you very much for taking the time to review our paper. We address your comments below and believe that our replies resolve your concerns.

 1. In your peer review you point out that “our conclusions seem premature because of possible selection bias“. It is unclear to us whether this refers to the selection of HTA reports or to the selection of primary studies into these HTA reports. Taking your argument in the first sense, we agree that our conclusions primarily refer to German HTA reports. It may well be that, due to different legal frameworks, HTA reports in other countries address other medical topics or address the same topics using other methods of information retrieval. To emphasize that these are the results of a German HTA agency, we have adapted the text accordingly.

**Change in the text: **instead of referring to HTA reports in general, the text now refers specifically to German HTA reports

 2. The issue of selection bias could also be understood in the sense of publication bias. The reviewer suspects that no difference between inclusion and exclusion of non-English sources was found, because relevant literature was not found by any of search approaches. According to the reviewer, this requires “an extensive search for grey literature, including non-peer reviewed articles, the types of articles that the authors have excluded (i.e., conferences abstracts, evidence syntheses, clinical study reports, or registry entries).“ As shown in Table 2, the information retrieval for IQWiG reports includes a number of databases (with a range of 3-23 per report). In addition, study registries were searched, reference lists screened, requests to authors were made and - for all reports but rapid reports - public hearings took place. Therefore, we assume the there is no issue with publication bias. Furthermore, the remaining risk of publication bias would be the nearly the same for both approaches, i.e. with or without non-English sources, because most conference abstracts, grey literature, and study reports will either be written in English or will be unusable for HTA purposes due to a lack of key study information.

**No change to the text**

 3. Furthermore, you suggest that the authors discuss the types of bias and their relative importance for the design of the study. From our point of view, it is highly likely that all the forms of bias mentioned had no influence on the changes to the conclusions. Especially if non-significant results tend to end up in non-English journals, this would have resulted in apparent differences between those HTA reports that included non-English sources and those that did not.

**No change to the text**

 Finally, you state that there is no motivation to include only reports prepared by the German HTA organisation. We disagree, because it requires detailed data to perform the reassessments using other language selection criteria. It would have required multinational cooperation and adequate funding to scale up the study to several HTA agencies and several languages. We also see no point in expanding the sample. Especially since, as mentioned in our discussion, major HTA agencies such as AHRQ, HAS and NICE also use some sort of language restriction in their reports.

**No change to the text**
